# Data-driven analysis using multiple self-report questionnaires to identify college students at high risk of depressive disorder

**DOI:** 10.1038/s41598-020-64709-7

**Published:** 2020-05-12

**Authors:** Bongjae Choi, Geumsook Shim, Bumseok Jeong, Sungho Jo

**Affiliations:** 1 School of Computing, Korea Advanced Institute of Science and Technology (KAIST), Daejeon, South Korea; 2 KAIST clinic Pappalardo center, Korea Advanced Institute of Science and Technology (KAIST), Daejeon, South Korea; 3 Graduate School of Medical Science and Engineering, Korea Advanced Institute of Science and Technology (KAIST), Daejeon, South Korea

**Keywords:** Health care, Computer science

## Abstract

Depression diagnosis is one of the most important issues in psychiatry. Depression is a complicated mental illness that varies in symptoms and requires patient cooperation. In the present study, we demonstrated a novel data-driven attempt to diagnose depressive disorder based on clinical questionnaires. It includes deep learning, multi-modal representation, and interpretability to overcome the limitations of the data-driven approach in clinical application. We implemented a shared representation model between three different questionnaire forms to represent questionnaire responses in the same latent space. Based on this, we proposed two data-driven diagnostic methods; unsupervised and semi-supervised. We compared them with a cut-off screening method, which is a traditional diagnostic method for depression. The unsupervised method considered more items, relative to the screening method, but showed lower performance because it maximized the difference between groups. In contrast, the semi-supervised method adjusted for bias using information from the screening method and showed higher performance. In addition, we provided the interpretation of diagnosis and statistical analysis of information using local interpretable model-agnostic explanations and ordinal logistic regression. The proposed data-driven framework demonstrated the feasibility of analyzing depressed patients with items directly or indirectly related to depression.

## Introduction

Depressive disorder is a prevalent but serious disorder. It is estimated that 17% of the population experiences depressive disorder at some stage in their lives^1^. The ongoing challenge in research is that patients’ symptoms and pathophysiology are too diverse. Patients with depressive disorder vary considerably in clinical presentation, processes, treatment responses, genetics, and neurobiology. The Diagnostic and Statistical Manual of Mental Disorders (DSM-5)^2^ describes nine MDD symptoms: (1) depressed mood or irritability, (2) decreased interest or pleasure, (3) significant weight change (5%) or change in appetite, (4) change in sleep, (5) change in activity, (6) fatigue or loss of energy, (7) guilt/worthlessness, (8) concentration problems, and (9) suicidality. To satisfy the criteria for depression, patients require five or more symptoms. The current diagnostic practice relies on professional medical expertise and time-consuming interviews that require the close cooperation of patients. Because of the inefficiency of diagnostic practice, self-report questionnaires have been used widely to screen for the presence and severity of depression. Although clinical self-report questionnaires have been designed to provide questions about various depression-related items listed by patients, the results are subjective because they rely on patients’ responses.

There are various approaches to solve these problems, but we focused only on the data driven approach in this study. This approach is a method that is spurred on by data, as opposed to being driven by mere intuition or personal experience. Despite the obvious advantages of the data-driven approach to clinical applications such as diagnosing depression, many clinical institutions have not begun using it thus far. There are several examples of the common, yet avoidable, downsides of the data-driven approach. First, larger and more detailed data does not always lead to larger and more detailed information. The curse of dimensionality^3^, which is that difficulty in analyzing data grows exponentially with the dimensionality, is a good example of this. It can be troublesome to extract relevant medical information from a large number of clinical datasets. These limitations could prevent long-term tracking of the patient’s state. Second, data acquisition in the data-driven approach is costly and time-consuming and should be designed well before the data are collected. Most methods do not consider modifying or adding data specification. For example, replacing an old scale with a new scale or adding a new item to a questionnaire form could prevent the use of existing data. These limits may prevent long-term tracking of the patient’s state. The last has been mentioned as the medical expert system of the Artificial Intelligence Winter^4^. The data-driven approach should provide a diagnostic decision-making process as well as providing diagnostic results. It should consist of the evidences, symptoms, statistical tests and other data all available to the clinician.

Several studies have been conducted to diagnose depressive disorder using data-driven approaches, relying on clinical questionnaires^5–9^. Lin *et al*.^5^ identified 12 risk predictive rules and the rule-based prognostic model to follow up on status of patients with depression. It is based on electronic health record containing Patient Health Questionnaire9 (PHQ-9)^10^. Dinga *et al*.^6^ predicted the naturalistic course of depression from clinical, psychological, and biological data via penalized logistic regression. The general limitations of clinical application based on the supervised predictive model is that collecting the labeled sample of patients is more costly and time-consuming relative to the unsupervised approaches because it needs professional medical expertise. Hybels *et al*.^7^ identified four clusters of 366 patients, aged 60 years old or over, with major depression using latent cluster analysis; however, it examined mainly depression severity. Similarly, Lamers *et al*.^8^ grouped patients into three clusters according to depression severity. Tokuda *et al*.^9^ performed multiple co-clustering for depressive disorder using a high-dimensional dataset consisting of fMRI, clinical questionnaire scores, and various biomarkers. However, these approaches focus primarily on identifying subtypes of depressive disorder through unsupervised cluster analysis. These types of models reduce a large number of data from individuals to smaller number of latent variables based on similarity. Because only similarity between data distributions is compared, clusters are likely to form based on factors that may not be crucial. For instance, we may want to categorize patients according to severity. However, the clusters might be trained based on other items such as age and gender. In addition, most cluster analysis requires prior knowledge of clusters or the number of clusters for good performance.

Extending these perspectives, this study developed a data-driven framework able to analyze depressive disorder using clinical surveys acquired from multiple questionnaire forms. This was achieved via state-of-the-art machine learning techniques including deep learning, multi-modal representation learning, and interpretability of the machine learning model. We proposed shared representation model between three questionnaire forms without paired observations. To overcome the limitations of the supervised and unsupervised approaches, we implemented the semi-supervised diagnostic method. In addition, the study addressed the limitations of the data-driven approach by providing an interpretation of diagnosis and the statistical analysis of information. Our method allowed clinicians to track the patient’s state in the long term, regardless of changes in questionnaire items.

## Results

### Participants

The participants comprised college students who had undergone health screening at KAIST Clinic Pappalardo Center from April 2013 to July 2017. In total, 14,929 participants participated 25,539 times, and 6,186 participants participated in the screenings more than once. Further, 856 out of 25,539 records (3.35%) had missing values. We used mean value imputation to complete the missing data. The KAIST Institutional Review Board approved the research protocol for the study. All research was performed in accordance with the relevant guidelines and regulations. Informed consent was obtained from all participants.

The questionnaire consisted of three types of items. First, the questionnaire contained demographic information such as age, sex, family members, and grade. Second, physical and mental health questions that were indirectly related to depression, such as smoking status, drinking status, sleep level, sleep regularity, and dependence on smart phones, were included in the questionnaire. Third, the questionnaire included items directly related to depression and mental illness: Beck Depression Inventory (BDI)^11^, General Anxiety Disorder 7 (GAD-7)^12^, Resilience Appraisals Scale (RAS)^13^, PHQ-9. As we updated the questionnaire forms, three different forms were used in the study. According to the acquisition period, we defined them as the Questionnaire Forms I, II and III. From April 2013 to March 2014, we collected 6,555 data using a questionnaire consisting of 44 items including the BDI (Questionnaire Form I)), and 5,618 data were acquired using Questionnaire Form II (38 items). We removed several items from the BDI and added the PHQ-9 and GAD-7 to Questionnaire Form II. In the remaining period, we collected 13,366 samples through Questionnaire Form III composed of 30 items. The total number of questionnaire items used in the study was 55. The detailed description of each questionnaire form is provided in the supplementary file.

We evaluated our results by analyzing the distribution of patient groups. The evaluation data consisted of two patient groups. The first group consisted of 106 participants screened using Questionnaire Form III to be at high risk for depressive disorder confirmed by a clinical psychologist’s telephone interviews. The second group consisted of previous survey responses from 66 depressed patients who visited the department of psychiatry at the university clinic.

### Study design

In this study, we developed a depression diagnosis and analysis system that able to overcome the limitations of the previous data-driven approach. We analyzed clinical surveys acquired from multiple questionnaire forms. Because each questionnaire form has different items and characteristics, we have to project them into a single representation to use a data-driven approach. This is called shared representation between multiple questionnaire forms. It can be learned through multi-modal learning. Modality refers to a particular way of doing or experiencing something. The modality of our study, the questionnaire responses, represents states of the patient for clinical application. Multi-modal representation learning is concerned with the problem of learning representations of data acquired from multiple modalities that facilitate extracting readily useful information when developing data-driven models^14^.

In this study, we performed multi-view learning, a special case of multi-modal learning, that aims to learn the shared representation between three different questionnaire forms which have the same modality but different views. Unfortunately, there are no paired observations between different questionnaire forms. In multi-view learning, for paired observations between different views, responses of the equivalent states of the participant in different forms, are necessary. However, these paired observations cannot be obtained naturally for clinical applications such as diagnosing depression. All questionnaire forms are designed to screen mental disorders, including depression, anxiety and attention deficit hyperactivity disorder (ADHD). Therefore, we can divide items of the questionnaire forms into common items, or those that are common to all questionnaires, and different items, the items that are not common. For instance, all of the Questionnaire Forms I, II, III contained the Pittsburgh Sleep Quality Index (PSQI)^15^, which measures the quality and patterns of sleep over a 1-month time interval. The common items of three questionnaire forms included the PSQI score. The BDI, only used in Questionnaire Form I, was one of the different items of Questionnaire Form I. In contrast, the different items of Questionnaire Forms II, III contained the PHQ-9 and GAD-7. Our proposed method learns shared representations between multiple questionnaire forms based on paired observations of common and different items.

After learning shared representation between multiple questionnaire forms, we proposed two data-driven diagnostic methods to diagnose depressive disorder; unsupervised and semi-supervised. We compared them with the conventional questionnaire-based depression diagnostics, which compare the items directly related to depression via cut-off points. From this point on, we call this diagnostic method the screening method. We used the BDI, PHQ-9, and GAD-7 to divide participants into three groups according to severity. The BDI and PHQ-9 are commonly used to diagnose depressive disorders. The GAD-7 measures the severity of anxiety, which is closely related to depressive disorders. The first group, Healthy, consisted of healthy participants with BDI scores of <10, PHQ-9 scores of <5, and GAD-7 scores of <5. The second group, Mild, consisted of participants with BDI scores of 10 to 15, PHQ-9 scores of 5 to 9, or GAD-7 scores of 5 to 9, classified as mildly depressed. Patients whose symptoms were severe than the mild depression group were placed in the third group, Severe, and showed BDI scores of>16, PHQ-9 scores of>10, or GAD-7 scores of>10. If a participant fulfilled both criteria of mild and severe groups, the one was categorized as severe group. For example, patients with a PHQ-9 score of 7 and GAD-7 score of 15 were classified into the Severe group based on GAD-7 scores. The baseline demographics and clinical characteristics of the participant groups diagnosed via the screening method are listed in Table 1.

**Table 1:** Demographics and clinical characteristics of participant groups.

	Healthy (n = 19,763)	Mild (n = 4,197)	Severe (n = 1,579)	Total (n = 25,539)
Female, n (%)	3,579 (18.1%)	1,093 (26.0%)	474 (30.0%)	5,146 (20.1%)
Age	23.2 ± 4.0	23.7 ± 4.1	24.2 ± 4.1	23.3 ± 4.0
BDI	3.2 ± 2.9	12.0 ± 1.6	21.2 ± 5.8	5.7 ± 6.2
PHQ-9	1.1 ± 1.3	6.1 ± 1.8	12.3 ± 3.9	2.6 ± 3.5
GAD-7	0.5 ± 0.9	3.5 ± 2.5	8.3 ± 4.7	1.5 ± 2.7

An unsupervised method was used to find inherent similarities between the participant groups. Clusters shared representations of questionnaire responses using a simple cluster analysis algorithm, the Gaussian mixture model. Clusters learned using unsupervised cluster analysis could form based on factors that may not be crucial because only similarities between data distributions was considered.

**Figure 1:**
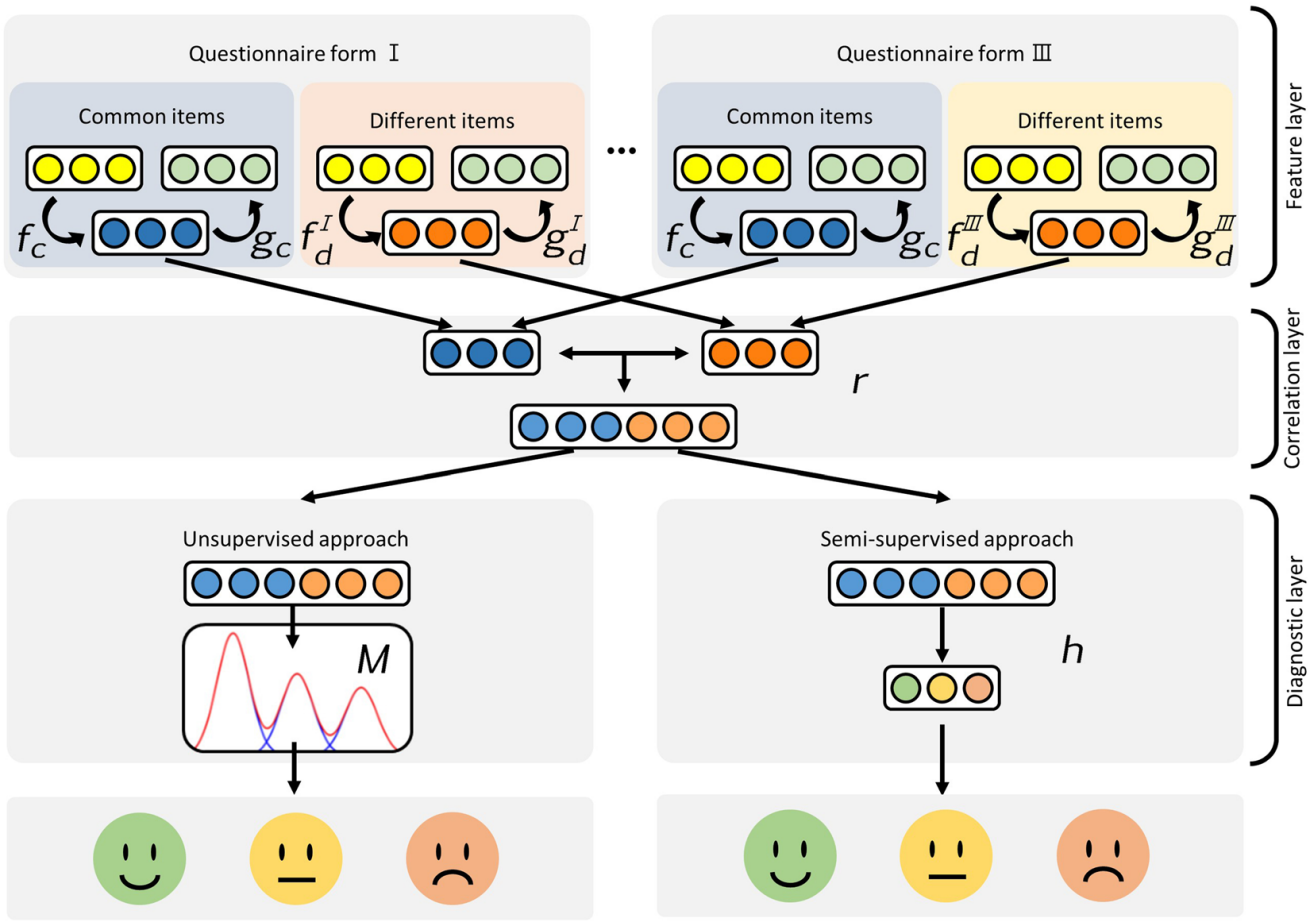
Schematic diagram of the proposed multi questionnaire representation learning model for depressive disorder diagnosis. The feature layer extracts the latent features from the questionnaire items using auto-encoders. The inward and outward arrows depict the encoding/decoding of the input features. The correlation layer projects the latent features of the common and different items into the shared representation using the canonical correlation between them. At the diagnostic layer, two approaches diagnose the participants based on shared representation, which is generated in the correlation layer. The unsupervised approach clusters participants into three groups using a Gaussian mixture. The semi-supervised approach classifies participants into three groups using another neural network.

Semi-supervised learning is a branch of machine learning techniques that uses both labeled and unlabeled data for training: typically a small number of labeled data with a large number of unlabeled data. Semi-supervised learning falls between unsupervised learning (without any labeled training data) and supervised learning (with completely labeled training data). In this study, we used semi-supervised methods to correct the characteristics of the unsupervised clustering that maximized only the differences between the participant groups. A small amount of diagnostic results using the conventional screening method helped to learn the actual characteristics of the participant groups. Figure 1 shows a schematic diagram of the proposed methods.

### Visualization of shared representation

**Figure 2:**
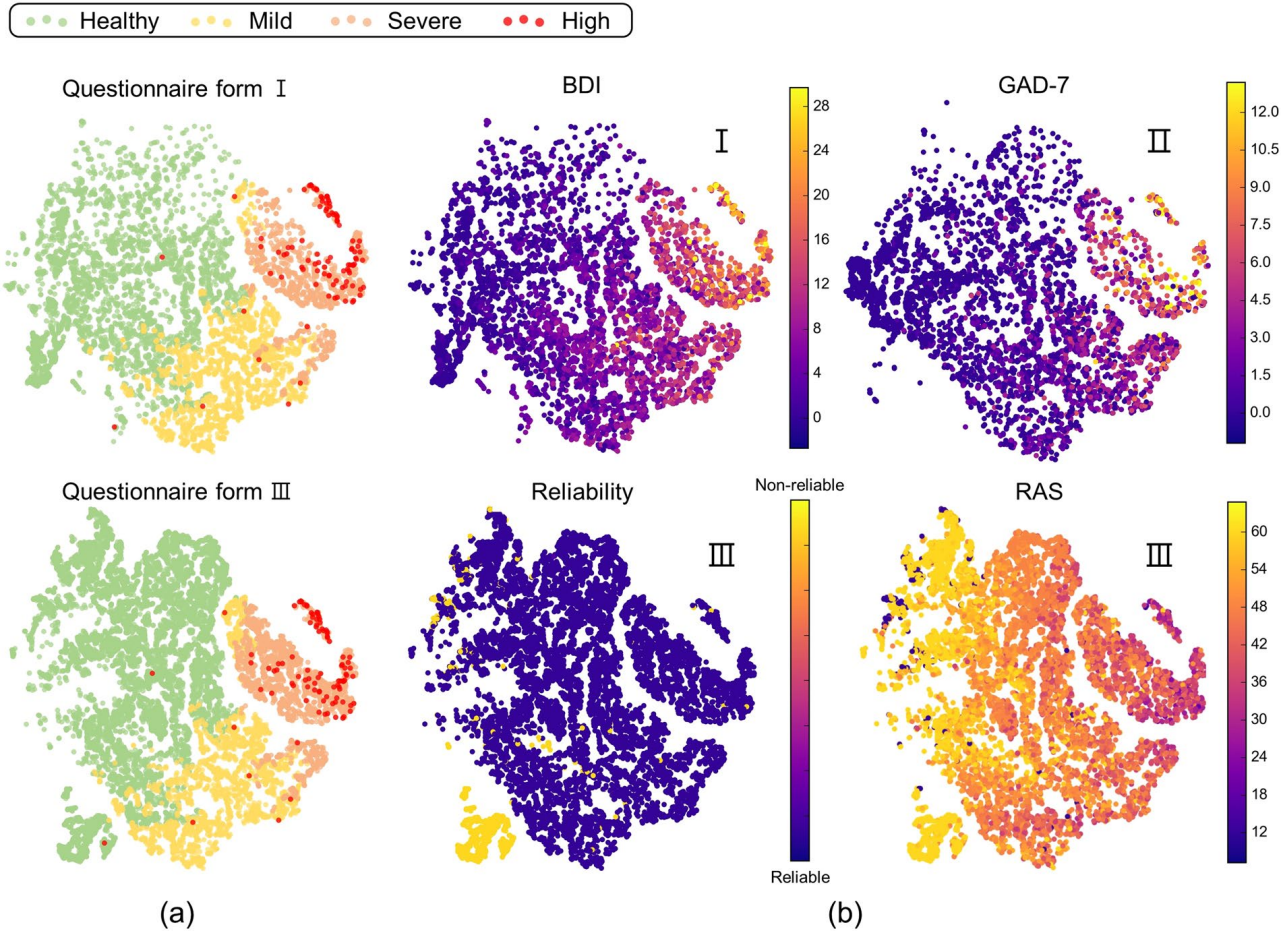
Visualization of learned shared representations. (**a**) Diagnostic results using the semi-supervised classification. The upper scatter plot is from Questionnaire Form I. The lower plot represents participants of Questionnaire Form III. Red marks are patients at high risk. (**b**) The BDI is from Questionnaire Form I. GAD-7 is from Questionnaire Form II. Response reliability is from Questionnaire Form III. RAS is from Questionnaire Form III.

Figure 2 shows visualization of learned shared representation. We extracted eight dimensional shared representations from each sample, then reduced them into two dimensions using t-distributed Stochastic Neighbor Embedding (t-SNE)^16^ which is widely used to visualize high dimensional data. Figure 2(a) shows the diagnostic results using the semi-supervised approach. It can be seen that the shared representation learned from two different questionnaire forms show similar distributions. It is also possible to see that the patients with depression are well clustered. Figure 2(b) illustrates the distribution of several items from different questionnaire forms. BDI and GAD-7, which positively correlate with depression, have high values concentrated in the severe depression area shown in Fig. 2(a). In contrast, the RAS, which negatively correlates with depression, tends to be low in the same region. We were also able to find that samples which have lower response reliability clustered in the lower left corner of scatter (yellow colored cluster in Fig. 2(b) Reliability). This means that most of non-reliable responses are not severely depressed patients. Non-reliable participants are participants who responded randomly, reduced, hid, or exaggerated their symptoms to the questionnaire. They are difficult to diagnose depression even with the proposed method. Based on these facts, we can indirectly see that shared representation have been well learned.

### Distribution of participants

**Figure 3:**
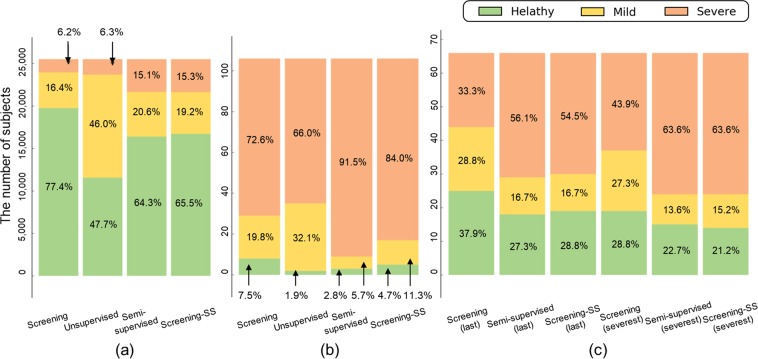
Distribution of groups according to diagnostic methods. (**a**) Training dataset. (**b**) 106 participants at high risk. (**c**) 66 depressed patients who visited the clinic.

Figure 3(a,b) show the distribution of all participants of the learning and those confirmed to be at high risk. In the training dataset, both unsupervised and semi-supervised approaches show the larger mild groups compared to the screening method. The unsupervised approach, in particular, set the severe group too narrow to focus on the large difference in feature distribution. As a result, it does not distinguish patients with depression compared to the screening method. Conversely, the semi-supervised approach was more successful in diagnosing depression. For patients at high risk, the diagnosis rates for the screening, unsupervised, and semi-supervised methods were 72.6%, 66.0%, and 91.5%, respectively.

However, as there was no healthy group included in the evaluation, it was difficult to determine whether the improvement in performance using the semi-supervised approach was real or a trivial result that occurred because of the increment of the predicted severe depression caused by lowering the cut-off point. To clarify this, we applied an additional diagnostic method, “Screening-SS,” as shown in in Fig. 3(a,b), which used new cut-off points that explained the semi-supervised approach using ordinal logistic regression. Detailed descriptions of the cut-off points can be found in subsection “ordinal logistic regression.” Because the diagnosis rate for “Screening-SS” was 84.0%, the semi-supervised approach showed superior performance but did not show a significant difference between approached (p > 0.05). However, when compared to the semi-supervised method, “Screening-SS” did not provide an explanation for decision-making processes, with the exception of the cut-off items. In the subsection “Interpretability,” we discuss the interpretability of the proposed method.

Figure 3(c) shows whether visits to the clinic by patients with depression could be predicted based on past questionnaires. Unlike in Fig. 3(b), which shows the current data, it was more difficult to diagnose patients based only on previous data. Figure 3(c) shows the distributions of the participants when the system only used data from the last questionnaire (labeled as “last”), and those for the severest cases in the previous questionnaires (labeled as “severest”). In both cases, the semi-supervised approach was superior to the screening method in predicting depression. The semi-supervised approach and “Screening-SS” showed similar performance in this patient group. Further, it was better to use the severest scores rather than the last scores to predict depression.

### Ordinal logistic regression

Ordinal logistic regression was performed to analyze the contribution of each questionnaire item to each diagnostic method. This method predicts an ordinal dependent variable given independent variables^17^. In this study, we used ordinal logistic regression to determine whether each questionnaire item was able to predict the diagnosis results. If each item predicted the outcome of the diagnosis appropriately, the approach considered the items and the correlations between them.

We analyzed the ordinal logistic regression model from two perspectives: slope *m* and x-intercept *a*. In logistic regression, there are several tests, such as likelihood ratio test, Wald test, and score test, to evaluate the goodness of fit of the model and the significance of dependent variables. In the current study, we used the Wald test, in which results are obtained by comparing the maximum likelihood estimate of the slope coefficient. For example, let us assume that an item is positively correlated with depression. The slope of the ordinal logistic regression model, which predicts the diagnosis results based on it, will be positive. In this case, if the slope of the ordinal logistic regression model for one diagnostic method is significantly larger relative to the slope of the model of the other diagnostic methods, the item is more significant in this diagnostic method than it is in other methods. In ordinal logistic regression, an x-intercept is the threshold between two groups. It is equivalent to the cut-off value for the screening method. By analyzing the pattern of the slope and x-intercept, we examined the differences between the three diagnostic methods.

**Figure 4:**
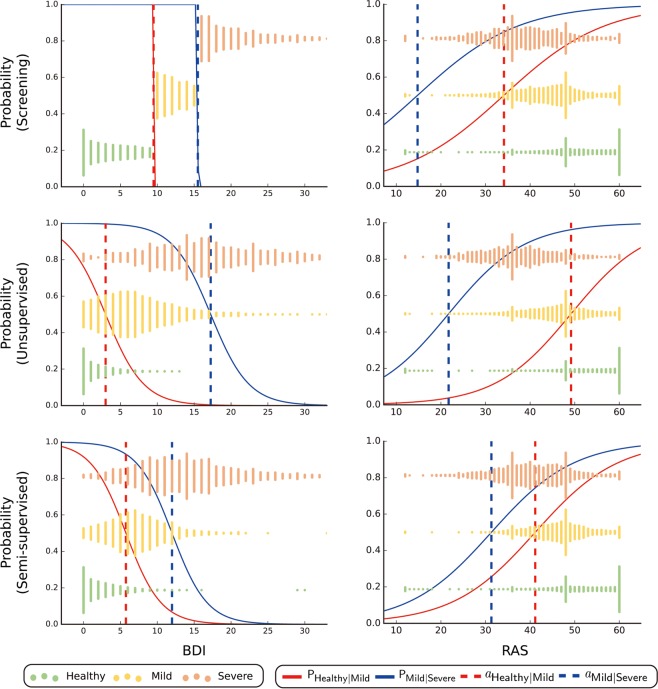
Results of ordinal logistic regression analysis. The left and right columns are the results of ordinal logistic regression with the BDI and RAS, respectively. The first row is the fitted model for the screening method. Similarly, the second and third rows show the unsupervised method and semi-supervised method, respectively. ${{\rm{P}}}_{{\rm{Healthy}}|{\rm{Mild}}}$ is the probability of belonging to Healthy. ${{\rm{P}}}_{{\rm{Mild}}|{\rm{Severe}}}$ is the probability that the depression is not Severe. ${a}_{{\rm{Healthy}}|{\rm{Mild}}}$ refers to the cut-off value between Healthy and Mild. ${a}_{{\rm{Mild}}|{\rm{Severe}}}$ indicates the cut-off value between Mild and Severe. The x-axis of the graph represents the value of the independent variables, and the y-axis is the probability of belonging to a particular group classified through ordinal logistic regression. Red solid lines depict the probability of being in the healthy group, and red dotted lines depict the cut-off value between the healthy group and patients with mild depression. Likewise, blue solid lines indicate the probability of being healthy or having mild depression, and blue dotted lines represent a cut-off value between mild and severe depression. The scatter is a histogram showing the distribution of groups diagnosed via each diagnostic method.

The significance of the total 55 features in three diagnostic methods was examined. Figure 4 shows two examples of ordinal logistic regression analysis. The left plot illustrates the ordinal logistic regression with the BDI, which is directly related to depression and used in the screening method. The slope fitted by ordinal logistic regression for the screening, unsupervised, and semi-supervised methods were 48.06, 0.39, and 0.42, respectively. The slope for the screening method was extremely steep, because the features were used directly in the screening method, and the cut-off values (9.50, 15.50) were almost closed to the criteria (10, 16) used in the screening method.

The right column is the RAS, which measures positive self-appraisals that buffer individuals from experiencing suicidal ideation. It is known to be negatively correlated with depression. The slope fitted by ordinal logistic regression for the screening, unsupervised, and semi-supervised methods were −0.09, −0.12, and −0.11, respectively. There were significant differences observed between the slopes (p < 0.05).

As mentioned in the subsection “Distribution of participants,” ordinal logistic regression based on the semi-supervised approach was used to calculate a new criterion for “Screening-SS” in Fig. 3. For the new cut-off points for Mild and Severe, fit values by ordinal logistic regression were (5.75, 12.00), (3.37, 6.43), and (2.14, 4.53) for the BDI, PHQ-9, and GAD-7, respectively.

**Figure 5:**
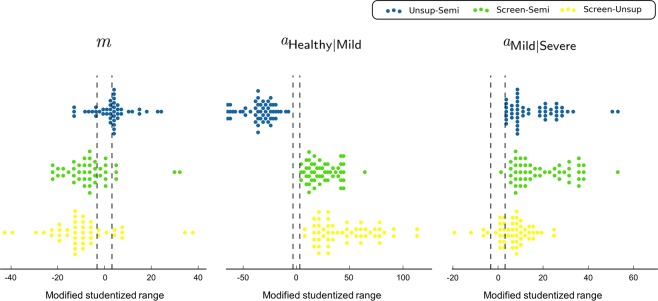
Visualization of significant differences between parameters. *m* is the slope coefficient for the fitted ordinal logistic regression model. ${a}_{{\rm{Healthy}}|{\rm{Mild}}}$ is the cut-off value between Healthy and Mild. ${a}_{{\rm{Mild}}|{\rm{Severe}}}$ indicates the cut-off value between Mild and Severe. The x-axis is the studentized range used in Tukey’s test multiplied by the sign of slope to consider the positive and negative correlations. The differences between the diagnostic methods were grouped by color. Dotted lines indicate critical values at the 0.05 level of significance for a two-sided test. Parameters located between the dotted lines indicate no statistical difference between two methods.

Figure 5 shows the significant difference between parameters based on the studentized range used in Tukey’s test multiplied by the sign of slope, to consider positive and negative correlations. In general, the slopes of the proposed data-driven diagnostic methods, the unsupervised and semi-supervised approaches, were steeper relative to that for the screening method. Therefore, both proposed methods considered each questionnaire item more thoroughly relative to the screening method. The slope parameters for 80.00% of items differed significantly between methods (p < 0.05). The proportions of the screening, unsupervised, and semi-supervised methods with the steepest slopes were 15.91%, 70.45%, and 13.64%, respectively. Exceptionally, items used directly used in the screening methods, such as the BDI, PHQ-9, and GAD-7, showed the steepest slopes in the screening method. With respect to the cut-off values, that for the unsupervised approach was generally wider relative to those for both the screening and semi-supervised methods, because it considered only the distribution of features as mentioned above. The cut-off values for Healthy and Mild in the unsupervised method were lower relative to both the screening and semi-supervised methods. The cut-off values for Mild and Severe in the unsupervised method was lower relative to that for the screening method but higher relative to that for the semi-supervised method. The ordinal logistic regression analysis results of the semi-supervised approach had a lower cut-off value relative to that of the screening approach. The detailed statistical analysis is provided in the supplementary file.

### Interpretability

In this study, we used the local interpretable model-agnostic explanations (LIME)^18^ to analyze causative relationships for decision making to verify that the proposed approach could be used to diagnose depressed patients with appropriate evidence. LIME is a method used to make arbitrary black box models locally explainable via sparse linear combinations around data that can already be explained.

To analyze the difference between the traditional screening diagnosis method and the proposed semi-supervised diagnosis method, we divided the patients who visited the clinic into three cases: Easy, Moderate, and Hard. The criterion for dividing these patients was whether the two diagnostic methods were able to categorize these depression patients as a Severe group. Case Easy was a set of patients which both the semi-supervised and screening methods successfully diagnosed or classified into a Severe group. The patients were categorized as case Moderate if the semi-supervised method predicted depression, but the screening method was unable to do so. Case Hard involved cases in which both the semi-supervised and screening methods were unable to predict severe depression. The cases for which the screening method predicted the depression, but the semi-supervised method failed to diagnose it did not exist. The difficulty of diagnosis ascended in the following order cases Easy, Moderate, and Hard. Among the 66 patients who visited the clinic for depression, 29, 13, and 24 were classified into each case, respectively.

**Figure 6:**
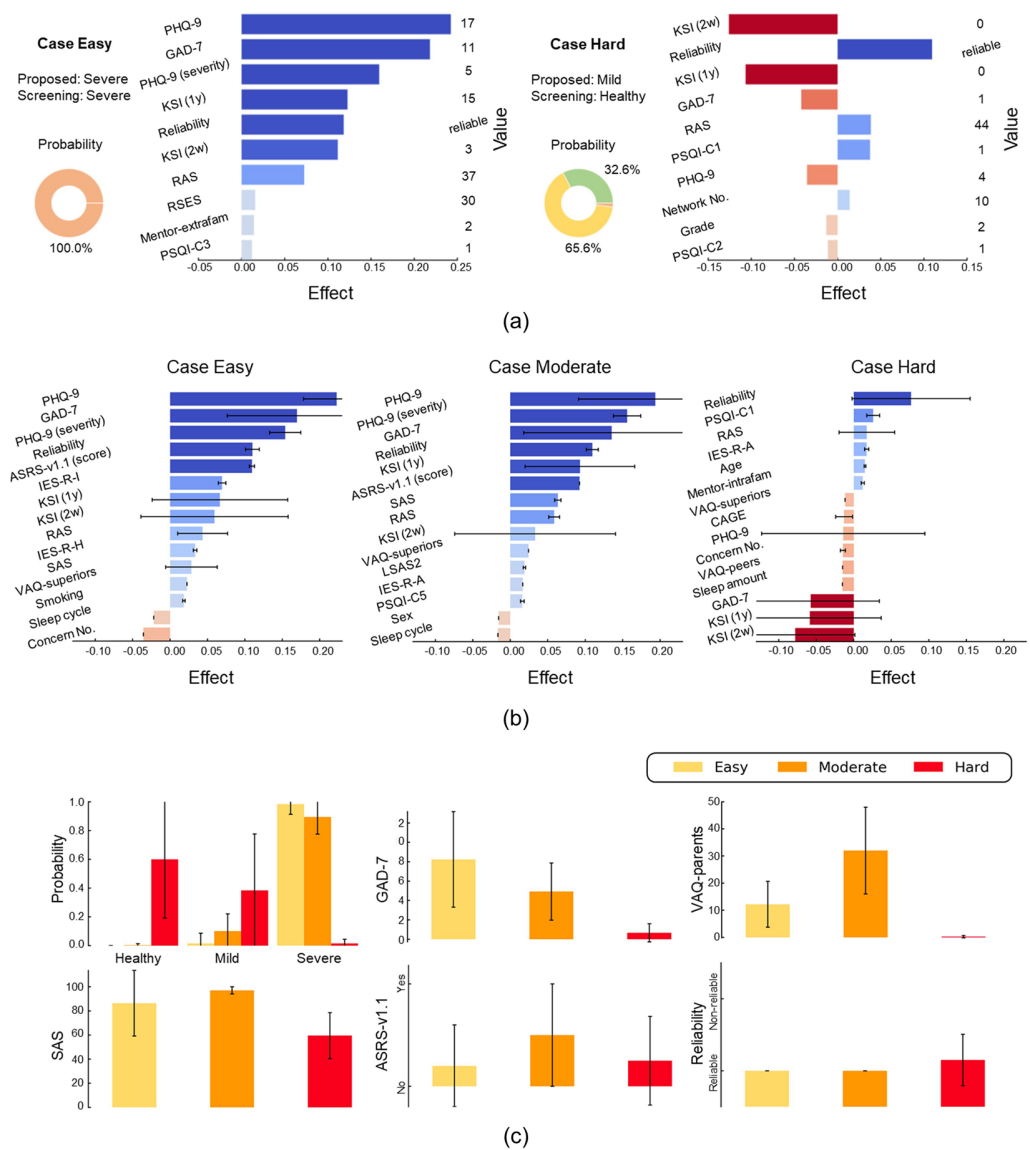
LIME analysis results for patients diagnosed via semi-supervised method. (**a**) Diagnostic example of patients using LIME. It shows probabilities predicted by the diagnostic method and describes values and the weighting of questionnaire items that support or oppose a diagnosis of severe depression. Positive weighting indicates that the questionnaire item supports the diagnosis of depression. Conversely, negative weighting is opposed to a diagnosis of depression. (**b**) Statistics in which questionnaire items affect the diagnosis of depression (top 15 items) for each case. (**c**) Statistics of diagnostic probabilities and questionnaire item values for each case. Abbreviations: KSI, KAIST Scale for Suicidal Ideation; RSES, Rosenberg Self-Esteem Scale; PSQI, Pittsburgh Sleep Quality Index; ASRS, Adult ADHD Self-Report Scale; IES-R, Impact of Event Scale-Revised; SAS, Smartphone Addiction Scale; LSAS, Liebowitz Social Anxiety Scale; VAQ, Verbal Abuse Questionnaire.

Figure 6 shows the LIME analysis results for the patients who were diagnosed via the semi-supervised method. Figure 6(a) shows examples of the diagnosis of patients who were analyzed using LIME. The left patient, which belonged to the case Easy, is a sample for which the proposed model successfully predicted depression with 100% probability as Severe. It shows that depression was identified because of high scores for the PHQ-9, GAD-7, and KAIST Scale for Suicidal Ideation (KSI)^19^, with low RAS scores. In the right sample, a patient in the case Hard category, the semi-supervised method diagnosed the patient as having mild depression. However, the screening method classified the patient into the healthy group. The proposed method diagnosed that this patient did not have severe depression because of low PHQ-9, GAD-7, and KSI scores. Conversely, there were some characteristics of severe depression such as low RAS score, high PSQI score, and high response reliability.

Figure 6(b) illustrates the weight of the questionnaire items in LIME analysis for each case. Case Easy on the left is a set of typically depressed patients who were successfully identified by both the proposed method and the traditional screening method. Values directly related to depression, such as PHQ-9, GAD-7, and KSI scores had high weighting. In addition, indirectly related items, such as Adult ADHD Self-Report Scale (ASRS-v1.1) scores^20^, Smartphone Addiction Scale (SAS) scores^21^, and RAS scores had high weighting, indicating that the semi-supervised method used them to diagnose depressed patients based on items similar to those in the decision-making process of clinical psychiatrists. The weight distribution of case Moderate in the middle was similar to that of case Easy but with subtle differences. This suggests that the proposed method classified patients through decision-making similar to case Easy, which considers the items that are not considered by the screening method. Case Hard on the right shows high negative weightings for the PHQ-9, GAD-7, and KSI, which were directly related to depression and had high weighting in cases Easy and Moderate. In other words, both diagnostic methods failed to predict depression because their responses were opposite to those for typical depression. Nevertheless, some indirectly related questionnaires such as the RAS, Impact of Event Scale-Revised (IES-R)^22^, and Verbal Abuse Questionnaire (VAQ)^23,24^ tend to support depression. In all three cases, the response reliability of questionnaire is the basis for a diagnosis of depressive disorder. This indicates that the diagnosis changed with changes in reliability, as most of the samples with low response reliability were healthy, as shown in Fig. 2(b). In the training dataset, there were 1,132 non-reliable responses, and 95.8% and 96.7% of non-reliable responses were for healthy participants in the screening and semi-supervised methods, respectively.

Figure 6(c) illustrates the statistics of diagnostic probability (upper left) and some questionnaire items (other) in each case. Patients in case Easy were highly likely to be diagnosed with more than mild depression. The uncertainty of diagnosis increases with the degree of difficulty of diagnosis. In the statistics for the questionnaire, the items that were directly correlated with depression, such as GAD-7 scores, tended to show higher values as the difficulty of diagnosis reduced. VAQ, SAS and ASRS-v1.1 scores, which are not directly related to depression but exert an indirect effect showed higher values in Moderate cases, relative to Easy cases, which indicate typical depression. We could therefore infer that the proposed method succeeded in diagnosing patients in Moderate cases based on these items. In contrast, as these values were not high in Hard cases, the proposed method failed to identify these patients. Regarding response reliability, Easy and Moderate cases did not have non-reliable responses, but Hard cases included some samples with low reliability.

## Discussion

The present study proposed a data-driven depression diagnostic method that aimed to overcome the limitations of data-driven clinical application. We solved the difficulty of data acquisition by making the use of multiple questionnaire forms. This reduced the cost and time required for the data-driven approach and allowed us to track patients regardless of changes in questionnaire forms. Various learning and regularization methods based on deep learning allowed us to generalize large, noisy, and high-dimensional data. We also provided an interpretation of diagnosis and the statistical analysis of information using LIME and ordinal logistic regression. Traditional data-driven approaches lack these points and have not been used widely.

The population of the dataset in this study held specific characteristic. Because it was composed of college students from a science and engineering-oriented university, the population was highly educated, young, and contained a high proportion of male participants relative to that in the wider population. For example, sex was hypothesized to be a significant predictor of BDI-II scores, as prevalence rates for depression in women is generally higher relative to that in men^25^. There is some evidence that education level is (negatively) related to depressive symptoms^26,27^. In contrast, some studies have shown that college students are vulnerable to depression, anxiety, and suicidal ideation^19,28^. For this reason, the cut-off values for the depression scales were adjusted for various purposes in various studies^29–31^. In general, cut-off points for items in questionnaires, such as the BDI, PHQ-9, and GAD-7, are determined by the sensitivity and specificity of the diagnosis. In particular, in the BDI, cut-off points were not clearly presented in the original article^11^. Various cut-off points were used for purposes such as prevalence and cost. We showed that the cut-off points for ordinal logistic regression based on the semi-supervised approach showed lower performance, but this was similar to the semi-supervised approach. Our proposed framework could suggest new cut-off values appropriate for the population of the dataset and the purpose of the study. It is not necessary to learn complex machine learning models and use the hardware system. However, the semi-supervised approach provides a diagnostic decision-making process as well as providing diagnostic results.

One of the most striking features of the clinical use of self-report questionnaires is that we should consider the possibility of invalid responses from patients^32^. Patients may not respond truthfully; they could reduce, hide, or exaggerate their symptoms in a socially acceptable way, which is known as social desirability bias. This problem could be measured via response reliability in the present study. It could also show the acquiescent response bias, non-acquiescent response bias, and random responses. Our results showed that the reliability of responses is one of the most influential factors in depression diagnosis. We found that most shared representations of unreliable responses were clustered into the healthy group, even with the proposed method. We examined the decision-making process of the proposed framework and confirmed that response reliability exerted a strong influence on the diagnosis of depression. Even if the responses were unreliable and the proposed framework failed to diagnose depressed patients, it could be used to analyze depressed patients with items indirectly related to depression.

This study was based only on clinical questionnaires without taking biological data, such as fMRI and EEG, into account. As the proposed model is based on a multi-modal model, it is able to extend the research to a model that considers questionnaire responses and biological data in future studies. It is possible to improve the model’s performance by mixing biological data with the questionnaire responses. This would be relatively simple, as questionnaire responses are easy to acquire in large numbers relative to biological data.

In summary, we proposed a novel data-driven attempt to diagnose depressive disorder based on clinical questionnaires acquired from multiple questionnaire forms. We analyzed 25,539 responses of 14,929 college students across three different questionnaire forms over four years. Multi-questionnaire representation learning were proposed to represent shared representations between multiple questionnaire forms without paired observations. We suggested the unsupervised and semi-supervised diagnostic methods based on multi-questionnaire representation, and compared them with a traditional diagnostic method for depression. The semi-supervised approach was superior to the other methods since it considered indirectly related items to depression such as ASRS-v1.1, SAS, and RAS scores. We also used the ordinal logistic regression and model-agnostic interpretation method to analyze the results of the proposed methods. The interpretation of diagnosis and statistical analysis of information increased the interoperability of depression diagnostic results. The current study helps clinicians to track the patient’s state in the long term, regardless of changes in questionnaire items, and have confidence in diagnostic decision making for depression.

## Methods

### Multi-questionnaire representation learning

We use canonical correlation analysis (CCA) and its deep learning extensions which are state-of-the-art representative techniques in multi-modal learning^33,34^. CCA learns shared representations of two views by finding the linear combinations of two views which have maximum correlation with each other^35^. Deep canonical correlation analysis (DCCA)^33^ is an extension of CCA that addresses the linear limitation of CCA by finding maximally linearly correlated non-linear transformations of two vectors using deep neural network. We also applied the autoencoder to our model. An autoencoder is a neural network that is trained to copy its input to its output^36^. Autoencoders are a deep learning and nonlinear version of principal component analysis which represents the underlying manifold of data. It generally improves deep learning models and DCCA also benefits from the autoencoder^34^.

First, we divided the items of each questionnaire into common items and different (non-common) items. Then, we were able to represent each questionnaire response as paired observations from two views, denoted $({c}_{1},{d}_{1}),\ldots ,({c}_{N},{d}_{N})$, where *N* is the total number of samples. We also denote the data matrices for each view by $C=[{c}_{1},\ldots ,{c}_{N}]$ and $D=[{d}_{1},\ldots ,{d}_{N}]$. For multi questionnaire representation learning, neural network *f*_*c*_ and *f*_*D*_ extract nonlinear latent features from the common items and different items, respectively. A *i*-th questionnaire response sample $({c}_{i},{d}_{i})$ from questionnaire form *k*_*j*_ is projected into a latent feature $({f}_{C}({c}_{i}),{f}_{D}^{{k}_{i}}({d}_{i}))$. The deep canonical correlation $r$ between the extracted features ${f}_{C}(C)$ and ${f}_{D}(D)$ is maximized.

1 $$\mathop{{\rm{m}}{\rm{i}}{\rm{n}}{\rm{i}}{\rm{m}}{\rm{i}}{\rm{z}}{\rm{e}}}\limits_{f,g,r}\,-\frac{\alpha }{L}{\rm{t}}{\rm{r}}({U}^{{\rm{T}}}{\rm{C}}{\rm{o}}{\rm{v}}(r({f}_{C}(C)),r({f}_{D}(D)))V)+\frac{\beta }{N}\mathop{\sum }\limits_{i=1}^{N}(\Vert {c}_{i}-{g}_{C}({f}_{C}({c}_{i})){\Vert }^{2}+\Vert {d}_{i}-{g}_{D}^{{k}_{i}}({f}_{D}^{{k}_{i}}({d}_{i})){\Vert }^{2})$$

2 $${\rm{s}}{\rm{u}}{\rm{b}}{\rm{j}}{\rm{e}}{\rm{c}}{\rm{t}}\,{\rm{t}}{\rm{o}}\,\,{{\rm{U}}}^{{\rm{T}}}{\rm{C}}{\rm{o}}{\rm{v}}({\rm{r}}({{\rm{f}}}_{{\rm{C}}}({\rm{C}})),{\rm{r}}({{\rm{f}}}_{{\rm{C}}}({\rm{C}}))){\rm{U}}={{\rm{V}}}^{{\rm{T}}}{\rm{C}}{\rm{o}}{\rm{v}}({\rm{r}}({{\rm{f}}}_{{\rm{D}}}({\rm{D}})),{\rm{r}}({{\rm{f}}}_{{\rm{D}}}({\rm{D}}))){\rm{V}}={\rm{I}}$$

where *U* and *V* are the CCA directions. *L* is the output dimension of deep canonical correlation *r*. The first term aims to maximize the canonical correlation. In contrast, the second term aims to minimize unsupervised reconstruction loss of the autoencoder. *α* and *β* are trade-off parameters to balance the correlation loss and unsupervised reconstruction loss, respectively. Because each questionnaire form shares the network ${f}_{C}$, the concatenated vector of $r({f}_{C}(C))$ and $r({f}_{D}(D))$ is the shared representation between multiple questionnaire forms.

We used a variant of an autoencoder called the ladder network for the deep learning framework in this study^37,38^. Several studies have shown that the additional lateral connections between the encoder and the decoder play an important role in both unsupervised and supervised learning. Encoder *f* and decoder *g* of ladder networks in the feature layer have 500, 500, 500, and 500 hidden nodes. A feed-forward network in correlation layer *r* has 500, 500, and 4 hidden nodes. The shared representation of questionnaire responses has eight dimensions.

### Unsupervised clustering and semi-supervised classification

In this study, unsupervised clustering was used to find inherent similarities between the participant groups. After pre-training each autoencoder with only the reconstruction loss, we fine-tuned the unsupervised approach with additional correlation loss to learn shared representations. Shared representations between the participant groups were clustered using a Gaussian mixture with full covariance. We repeated standard k-means clustering ten times in the latent space to obtain initial centroids of Gaussian mixture M. In the unsupervised approach, *α* and *β* were set at 1 and 1000, respectively.

Semi-supervised learning is obtained by adding the following supervised loss to Eq. (1).

3 $$\mathop{{\rm{m}}{\rm{i}}{\rm{n}}{\rm{i}}{\rm{m}}{\rm{i}}{\rm{z}}{\rm{e}}}\limits_{f,r,h}\,-\frac{\gamma }{N}\mathop{\sum }\limits_{i=1}^{N}\,\log \,{\rm{P}}(h(r({f}_{C}({c}_{i})),r({f}_{D}^{{k}_{i}}({d}_{i})))={t}_{i})$$

where ${t}_{i}$ is the desired group for a *i*-th response sample. *γ* is a trade-off parameters to balance the supervised loss and other loss.

We randomly sampled a total of 300 samples, 100 from each group, which were classified based on the screening method. Semi-supervised learning was performed using pseudo-labels. The semi-supervised approach has an additional single layer feedforward network *h* after the correlation layer, which represents shared representations, to classify the participants into three groups. Similar to unsupervised clustering, each autoencoder is pre-trained with only reconstruction loss, and we performed fine-tuning that optimized the joint loss of the reconstruction, correlation, and supervised loss. In the semi-supervised approach, *α*, *β*, and *γ* were set at 10, 10, and 1, respectively. We empirically set trade-off parameters, *α*, *β*, and *γ* since the correlation, unsupervised reconstruction, and supervised losses have different scales and priorities. We performed z-score normalization for each item. As the dataset contained missing values for several items, we filled the missing values with the mean value of the item, zero. We used dropout regularization^39^ with probability of 0.05 for the input values. The autoencoder with dropout improves both the regularization of model and the performance of imputation^40^. The loss of the network was minimized using the Adam gradient descent algorithm with a learning rate of 0.0001^41^. We set a mini-batch size of 5,000 because the optimization of correlation loss requires a sufficiently large mini-batch, which contains enough information to estimate covariance^34^. The epochs of pre-training and fine-tuning were 2,000 and 5,000, respectively. We implemented the neural networks using PyTorch^42^, and trained on a NVIDIA GeForce GTX TITAN X Pascal. The pseudo code of the proposed model is provided in the supplementary file.

### Statistical analyses

We performed ordinal logistic regression analysis to investigate the effect of each item on each diagnostic method using the ‘ordinal’ R package^43^. We fitted the ordinal logistic regression model to predict the diagnostic results *y* with a single questionnaire item *x*.

4 $$\begin{array}{ccc}\log \,{{\rm{P}}}_{{\rm{H}}{\rm{e}}{\rm{a}}{\rm{l}}{\rm{t}}{\rm{h}}{\rm{y}}|{\rm{M}}{\rm{i}}{\rm{l}}{\rm{d}}} & = & \log \,\frac{{{\rm{P}}}_{{\rm{H}}{\rm{e}}{\rm{a}}{\rm{l}}{\rm{t}}{\rm{h}}{\rm{y}}}}{{{\rm{P}}}_{{\rm{M}}{\rm{i}}{\rm{l}}{\rm{d}}}\,+\,{{\rm{P}}}_{{\rm{S}}{\rm{e}}{\rm{v}}{\rm{e}}{\rm{r}}{\rm{e}}}}\,\,\,\,\,\,=\,\,\,\,{b}_{{\rm{H}}{\rm{e}}{\rm{a}}{\rm{l}}{\rm{t}}{\rm{h}}{\rm{y}}|{\rm{M}}{\rm{i}}{\rm{l}}{\rm{d}}}-mx,\\ \log \,{{\rm{P}}}_{{\rm{M}}{\rm{i}}{\rm{l}}{\rm{d}}|{\rm{S}}{\rm{e}}{\rm{v}}{\rm{e}}{\rm{r}}{\rm{e}}} & = & \log \,\frac{{{\rm{P}}}_{{\rm{H}}{\rm{e}}{\rm{a}}{\rm{l}}{\rm{t}}{\rm{h}}{\rm{y}}}\,+\,{{\rm{P}}}_{{\rm{M}}{\rm{i}}{\rm{l}}{\rm{d}}}}{{{\rm{P}}}_{{\rm{S}}{\rm{e}}{\rm{v}}{\rm{e}}{\rm{r}}{\rm{e}}}}\,\,\,\,=\,\,\,\,{b}_{{\rm{M}}{\rm{i}}{\rm{l}}{\rm{d}}|{\rm{S}}{\rm{e}}{\rm{v}}{\rm{e}}{\rm{r}}{\rm{e}}}-mx,\\ y & = & \{\begin{array}{cccccc}{\rm{H}}{\rm{e}}{\rm{a}}{\rm{l}}{\rm{t}}{\rm{h}}{\rm{y}}, & {\rm{i}}{\rm{f}} &  & mx & \le  & {b}_{{\rm{H}}{\rm{e}}{\rm{a}}{\rm{l}}{\rm{t}}{\rm{h}}{\rm{y}}|{\rm{M}}{\rm{i}}{\rm{l}}{\rm{d}}}\\ {\rm{M}}{\rm{i}}{\rm{l}}{\rm{d}}, & {\rm{i}}{\rm{f}}\,\,\,{b}_{{\rm{H}}{\rm{e}}{\rm{a}}{\rm{l}}{\rm{t}}{\rm{h}}{\rm{y}}|{\rm{M}}{\rm{i}}{\rm{l}}{\rm{d}}} &  <  & mx & \le  & {b}_{{\rm{M}}{\rm{i}}{\rm{l}}{\rm{d}}|{\rm{S}}{\rm{e}}{\rm{v}}{\rm{e}}{\rm{r}}{\rm{e}}}\\ {\rm{S}}{\rm{e}}{\rm{v}}{\rm{e}}{\rm{r}}{\rm{e}}, & {\rm{i}}{\rm{f}}\,\,\,{b}_{{\rm{M}}{\rm{i}}{\rm{l}}{\rm{d}}|{\rm{S}}{\rm{e}}{\rm{v}}{\rm{e}}{\rm{r}}{\rm{e}}} &  <  & mx &  & \end{array}\end{array}$$

The ordinal logistic regression basically provides estimates of slope *m* and y-intercept *b*. However, our interests in this study were not y-intercepts *b* but included x-intercepts *a*; ${a}_{{\rm{Healthy}}|{\rm{Mild}}}$ indicates the cut-off value between the Healthy and Mild group. Similarly, ${a}_{{\rm{Mild}}|{\rm{Severe}}}$ was used to distinguish between mild and severe depression. We approximated the mean and standard error of x-intercepts *a* using first order Taylor expansion^44^.

5 $$\hat{a}={\rm{E}}[a]={\rm{E}}\left[\frac{b}{m}\right]\approx \frac{\hat{b}}{\hat{m}}$$

6 $${\sigma }_{\hat{a}}={\rm{S}}{\rm{E}}[a]=\sqrt{\frac{1}{n}{\rm{V}}{\rm{a}}{\rm{r}}[{\rm{a}}]}=\sqrt{\frac{1}{n}{\rm{V}}{\rm{a}}{\rm{r}}\left[\frac{b}{m}\right]}\approx \sqrt{\frac{{\hat{b}}^{2}}{{\hat{m}}^{2}}\left[\frac{{\sigma }_{\hat{b}}^{2}}{{\hat{b}}^{2}},-,2,\frac{{\sigma }_{\hat{m}\hat{b}}^{2}}{\hat{m}\hat{b}},+,\frac{{\sigma }_{\hat{m}}^{2}}{{\hat{m}}^{2}}\right]}$$

We assumed that diagnosis results from three different approaches were independent. We compared regression coefficients between models^45,46^. Differences between regression coefficients were tested using one-way ANOVA and post-hoc analysis was performed using Turkey’s test.

## Supplementary information

Supplementary information

Supplementary information2

## Data Availability

Due to potentially identifying information, the data that support the findings of this study are not publicly available, but can be obtained on reasonable request from the corresponding author with the permission of the KAIST Institutional Review Board.
