# Supplementary material for: Data-driven analysis using multiple self-report questionnaires to identify college students at high risk of depressive disorder
Source: Sci Rep. 2020 May 12;10:7867. doi: 10.1038/s41598-020-64709-7 (PMC7217968; doi:10.1038/s41598-020-64709-7)
Supplement: Supplementary file 1 — Supplementary information [file 41598_2020_64709_MOESM1_ESM.pdf]

Data-driven analysis using multiple self-report questionnaires to identify college students at high risk of depressive disorder

Bongjae Choi[1], **Geumsook Shim**[2], **Bumseok Jeong**[2, 3, *], **and Sungho Jo**[1,*]

[1]School of Computing, Korea Advanced Institute of Science and Technology (KAIST), Daejeon, South Korea
[2]KAIST clinic Pappalardo center, Korea Advanced Institute of Science and Technology (KAIST), Daejeon, South Korea
[3]Graduate School of Medical Science and Engineering, Korea Advanced Institute of Science and Technology (KAIST), Daejeon, South Korea
[*]Corresponding authors: B. J. (bs.jeong@kaist.ac.kr) and S. J. (shjo@kaist.ac.kr)

ABSTRACT

Depression diagnosis is one of the most important issues in psychiatry. Depression is a complicated mental illness that varies in symptoms and requires patient cooperation. In the present study, we demonstrated a novel data-driven attempt to diagnose depressive disorder based on clinical questionnaires. It includes deep learning, multi-modal representation, and interpretability to overcome the limitations of the data-driven approach in clinical application. We implemented a shared representation model between three different questionnaire forms to represent questionnaire responses in the same latent space. Based on this, we proposed two data-driven diagnostic methods; unsupervised and semi-supervised. We compared them with a cut-off screening method, which is a traditional diagnostic method for depression. The unsupervised method considered more items, relative to the screening method, but showed lower performance because it maximized the difference between groups. In contrast, the semi-supervised method adjusted for bias using information from the screening method and showed higher performance. In addition, we provided the interpretation of diagnosis and statistical analysis of information using local interpretable model-agnostic explanations and ordinal logistic regression. The proposed data-driven framework demonstrated the feasibility of analyzing depressed patients with items directly or indirectly related to depression.

Supplementary information

Domain	Notation	Description
Data	N	Number of responses
	N_L	Number of responses with label
	m	Mini-batch size
	c_i	Common items of i-th questionnaire response
	d_i	Different items of i-th questionnaire response
	k_i	Questionnaire form of i-th questionnaire response
	x_i	i-th questionnaire response (c_i, d_i) from questionnaire form k_i
	y_i	Diagnostic result of i-th questionnaire response
	t_i	Desired group for i-th questionnaire response
	q	Questionnaire form q
	C	Common items of all questionnaire responses
	D^q	Different items of questionnaire responses from questionnaire form q
	D	Different items of all questionnaire responses
	X	Questionnaire responses
	X_L	Questionnaire responses with label
	Y	Diagnostic results
	Q	Questionnaire forms
Model	f_C	Encoder network for common items from all questionnaire forms
	g_C	Decoder network for common items from all questionnaire forms
	f_D^q	Encoder network for different items from questionnaire form q
	g_D^q	Decoder network for different items from questionnaire form q
	$f_C(C)$	Latent variables for common items from all questionnaire forms
	$f_D(D)$	Latent variables for different items from all questionnaire forms
	r	Deep canonical correlation network
	L	Output dimension of r
	U	CCA directions for common items
	V	CCA directions for different items
	M	Gaussian mixture for unsupervised clustering
	h	Classification network for semi-supervised classification
	α	Trade-off parameter for canonical correlation loss
	β	Trade-off parameter for unsupervised reconstruction loss
	γ	Trade-off parameter for supervised loss

Table S1. Notation for multi-questionnaire representation learning

Algorithm S1: Unsupervised pre-training of autoencoder

Procedure PretrainAutoencoder(R)

  Input: Items R

  Output: Encoder f, decoder g

  Initialize f and g

  for *each epoch* **do**

    for *each batch* **do**

      Sample a mini-batch of m responses $\{r_1, \ldots, r_m\}$ from R

      $\tilde{r}_i \leftarrow Dropout(r_i)$

      $h_i \leftarrow f(\tilde{r}_i)$

      $\hat{r}_i \leftarrow g(h_i)$

      $L_R \leftarrow \frac{1}{m} \sum_{i=1}^{m} (\left\| r_i - \hat{r}_i \right\|^2)$

      Calculate gradient of L_R w.r.t. parameters of f and g

      Update parameters by taking a gradient step

Algorithm S2: Unsupervised clustering of multi-questionnaire representations

Input: Questionnaire responses X
Output: Diagnostic results Y

/* Pre-train autoencoders */

$f_C, g_C \leftarrow$ PretrainAutoencoder(C)
for *each questionnaire form* q **do**
  $f_D^q, g_D^q \leftarrow$ PretrainAutoencoder(D^q)

/* Fine-tune unsupervised approach */

Initialize correlation network r
for *each epoch* **do**
  for *each batch* **do**
    Sample a mini-batch of m responses $\{(c_1, d_1, k_1), \ldots, (c_m, d_m, k_m)\}$ from X
    $\tilde{c}_i \leftarrow Dropout(c_i)$
    $\tilde{d}_i \leftarrow Dropout(d_i)$
    Calculate unsupervised reconstruction loss L_R
    $L_R \leftarrow \frac{1}{m}\sum_{i=1}^{m}\left(\left\|c_i - g_C\left(f_C\left(\tilde{c}_i\right)\right)\right\|^2 + \left\|d_i - g_D^{k_i}\left(f_D^{k_i}\left(\tilde{d}_i\right)\right)\right\|^2\right)$
    Calculate canonical correlation loss L_C
    $\Sigma_{CC} \leftarrow \mathrm{Cov}\left(r\left(f_C\left(\tilde{C}\right)\right), r\left(f_C\left(\tilde{C}\right)\right)\right)$
    $\Sigma_{DD} \leftarrow \mathrm{Cov}\left(r\left(f_D\left(\tilde{D}\right)\right), r\left(f_D\left(\tilde{D}\right)\right)\right)$
    $\Sigma_{CD} \leftarrow \mathrm{Cov}\left(r\left(f_C\left(\tilde{C}\right)\right), r\left(f_D\left(\tilde{D}\right)\right)\right)$
    $L_C \leftarrow -\frac{1}{L}\mathrm{tr}\left(U^\top \Sigma_{CD} V\right)$ subject to $U^\top \Sigma_{CC} U = V^\top \Sigma_{DD} V = I$
    $T \leftarrow \Sigma_{CC}^{-\frac{1}{2}} \Sigma_{CD} \Sigma_{DD}^{-\frac{1}{2}}$
    $L_C \leftarrow -\frac{1}{L}\sqrt{\mathrm{tr}\left(T^\top T\right)}$
    Calculate joint loss L of reconstruction and correlation loss
    $L \leftarrow \alpha L_C + \beta L_R$
    Calculate gradient of L w.r.t. parameters of f, g and r
    Update parameters by taking a gradient step

/* Unsupervised clustering based on multi-questionnaire representations */

Extract shared representation $\left(r(f_C(c_i)), r(f_D^{k_i}(d_i)\right)$ for all questionnaire responses x_i
Initial centroids of Gaussian mixture M using k-means clustering
Estimate centroids and full covariance of M with EM algorithm

/* Unsupervised diagnostic results */

for *each response* x_i **do**
  $y_i \leftarrow M\left(r(f_C(c_i)), r(f_D^{k_i}(d_i)\right)$

Algorithm S3: Semi-supervised classification of multi-questionnaire representations

Input: Questionnaire responses X, Questionnaire responses with label X_L
Output: Diagnostic results Y

`/* Pre-train autoencoders */`

$f_C, g_C \leftarrow$ PretrainAutoencoder(C)
for *each questionnaire form q* **do**
  $f_D^q, g_D^q \leftarrow$ PretrainAutoencoder(D^q)

`/* Fine-tune semi-supervised approach */`

Initialize correlation network r
for *each epoch* **do**
  for *each batch* **do**
    Sample a mini-batch of m responses, $\{(c_1, d_1, k_1), \ldots, (c_m, d_m, k_m)\}$ from X
    $\tilde{c}_i \leftarrow Dropout(c_i)$
    $\tilde{d}_i \leftarrow Dropout(d_i)$
    Calculate unsupervised reconstruction loss L_R
    $L_R \leftarrow \frac{1}{m} \sum_{i=1}^{m} \left(\left\| c_i - g_C\left(f_C\left(\tilde{c}_i\right)\right) \right\|^2 + \left\| d_i - g_D^{k_i}\left(f_D^{k_i}\left(\tilde{d}_i\right)\right) \right\|^2 \right)$
    Calculate canonical correlation loss L_C
    $\Sigma_{CC} \leftarrow \mathrm{Cov}\left(r\left(f_C\left(\tilde{C}\right)\right), r\left(f_C\left(\tilde{C}\right)\right)\right)$
    $\Sigma_{DD} \leftarrow \mathrm{Cov}\left(r\left(f_D\left(\tilde{D}\right)\right), r\left(f_D\left(\tilde{D}\right)\right)\right)$
    $\Sigma_{CD} \leftarrow \mathrm{Cov}\left(r\left(f_C\left(\tilde{C}\right)\right), r\left(f_D\left(\tilde{D}\right)\right)\right)$
    $L_C \leftarrow -\frac{1}{L} \mathrm{tr}\left(U^{\top} \Sigma_{CD} V\right)$ subject to $U^{\top} \Sigma_{CC} U = V^{\top} \Sigma_{DD} V = I$
    $T \leftarrow \Sigma_{CC}^{-\frac{1}{2}} \Sigma_{CD} \Sigma_{DD}^{-\frac{1}{2}}$
    $L_C \leftarrow -\frac{1}{L} \sqrt{\mathrm{tr}\left(T^{\top} T\right)}$
    Calculate supervised loss L_S
    Take all responses $\left\{(c_1, d_1, k_1, t_1), \ldots, (c_{N_L}, d_{N_L}, k_{N_L}, t_{N_L})\right\}$ from X_L
    $\tilde{c}_i \leftarrow Dropout(c_i)$
    $\tilde{d}_i \leftarrow Dropout(d_i)$
    $L_S \leftarrow -\frac{1}{N_L} \sum_{i=1}^{N_L} \log \mathrm{P}\left(h\left(r\left(f_C\left(\tilde{c}_i\right)\right), r\left(f_D^{k_i}\left(\tilde{d}_i\right)\right)\right) = t_i\right)$
    Calculate joint loss L of reconstruction, correlation, and supervised loss
    $L \leftarrow \alpha L_C + \beta L_R + \gamma L_S$
    Calculate gradient of L w.r.t. parameters of f, g, r, and h
    Update parameters by taking a gradient step

`/* Semi-supervised diagnostic results */`

for *each response x_i* **do**
  $y_i \leftarrow h\left(r(f_C(c_i)), r(f_D^{k_i}(d_i)\right)$
